# Improving early diagnosis of pulmonary infections in patients with febrile neutropenia using low-dose chest computed tomography

**DOI:** 10.1371/journal.pone.0172256

**Published:** 2017-02-24

**Authors:** M. G. Gerritsen, M. J. Willemink, E. Pompe, T. van der Bruggen, A. van Rhenen, J. W. J. Lammers, F. Wessels, R. W. Sprengers, P. A. de Jong, M. C. Minnema

**Affiliations:** 1 Department of Haematology, University Medical Center Utrecht Cancer Center, Utrecht, the Netherlands; 2 Department of Haematology, Academic Medical Center, University of Amsterdam, Amsterdam, the Netherlands; 3 Department of Radiology, University Medical Center Utrecht, Utrecht, the Netherlands; 4 Department of Respiratory Medicine, University Medical Center Utrecht, Utrecht, the Netherlands; 5 Department of Medical Microbiology, University Medical Center Utrecht, Utrecht, the Netherlands; 6 Department of Radiology, Meander Medical Center, Amersfoort, the Netherlands; University of California Los Angeles David Geffen School of Medicine, UNITED STATES

## Abstract

We performed a prospective study in patients with chemotherapy induced febrile neutropenia to investigate the diagnostic value of low-dose computed tomography compared to standard chest radiography. The aim was to compare both modalities for detection of pulmonary infections and to explore performance of low-dose computed tomography for early detection of invasive fungal disease. The low-dose computed tomography remained blinded during the study. A consensus diagnosis of the fever episode made by an expert panel was used as reference standard. We included 67 consecutive patients on the first day of febrile neutropenia. According to the consensus diagnosis 11 patients (16.4%) had pulmonary infections. Sensitivity, specificity, positive predictive value and negative predictive value were 36%, 93%, 50% and 88% for radiography, and 73%, 91%, 62% and 94% for low-dose computed tomography, respectively. An uncorrected McNemar showed no statistical difference (*p* = 0.197). Mean radiation dose for low-dose computed tomography was 0.24 mSv. Four out of 5 included patients diagnosed with invasive fungal disease had radiographic abnormalities suspect for invasive fungal disease on the low-dose computed tomography scan made on day 1 of fever, compared to none of the chest radiographs. We conclude that chest radiography has little value in the initial assessment of febrile neutropenia on day 1 for detection of pulmonary abnormalities. Low-dose computed tomography improves detection of pulmonary infiltrates and seems capable of detecting invasive fungal disease at a very early stage with a low radiation dose.

## Introduction

Neutropenic fever is one of the most important complications in cancer patients.[1] It is critical to rapidly localize infections and identify the organism involved, especially for fungal infections, which need a specific treatment approach. Despite a standard diagnostic workup including chest radiograph (CXR) and microbiological screening, no focus is identified in up to 44% of patients.[2] This can partly be explained by the low sensitivity of radiographs for diagnosing pulmonary infections in neutropenic patients.[3] Acquiring CXRs in respiratory asymptomatic patients with febrile neutropenia is therefore controversial. The ESMO guidelines recommend performance of a CXR in every neutropenic patient with fever, whereas the IDSA guidelines suggest its use only in patients with respiratory signs or symptoms.[1, 4]

In febrile neutropenia a substantial part (~10%) of infections is caused by invasive fungal disease (IFD).[5] IFD usually presents as a pulmonary infection, which is rarely visible on CXR and is therefore often missed in the early phase of neutropenic fever. Notably, a delayed start of appropriate treatment has a negative impact on clinical outcome.[6]

In order to improve the early detection of pulmonary infiltrates in febrile neutropenia, other imaging tools have been investigated as an alternative to CXR. High-resolution computed tomography (HRCT) improved pulmonary focus detection: pulmonary abnormalities were found in 60% of the neutropenic patients with persistent fever (>48 hours) and a normal CXR.[7] Furthermore, direct initiation of effective antifungal treatment after the early detection of a halo sign on HRCT images, had a positive effect on treatment response and survival in case of IFD, emphasizing the importance of early diagnosis.[8] However, due to costs and high radiation doses of approximately 7 mSv average, HRCT scanning is usually not incorporated in the initial febrile neutropenia workup.[9]

Another imaging modality that could be used for the evaluation of febrile neutropenia is low-dose CT scanning (LDCT). LDCT is performed with low mean radiation doses below 1.5 mSv and without the use of contrast. Two studies comparing LDCT to CXR in patients with persistent febrile neutropenia demonstrated an increased detection of pulmonary abnormalities.[10, 11] Therefore, we hypothesized that LDCT already acquired on day 1 of febrile neutropenia would improve detection of pulmonary infections. The primary aim of our study was to compare the diagnostic performance of LDCT and 2-view CXR for early detection of pulmonary infiltrates. The secondary aim was to explore its performance for early IFD detection.

## Materials and methods

The study was approved by the local institutional review board (approval number NL41415.041.12) of the university medical center Utrecht. Written informed consent was obtained from all patients. This prospective study was conducted at the haematology ward of the university medical center Utrecht, a tertiary care oncology center, between March 2013 and December 2014.

### Subjects

Patients with febrile neutropenia treated with intensive chemotherapy for haematological malignancies or receiving an autologous or myelo-ablative allogeneic stem cell transplant (SCT) were eligible for inclusion. Febrile neutropenia was defined as a single temperature measurement of ≥38.3°C or a temperature of ≥38°C for more than 1 hour, accompanied by an absolute neutrophil count of <0.5×10^9^/L or a neutrophil count <1.0×10^9^/L with a predicted decline to <0.5×10^9^/L within 3 days.

Patients were excluded in case of a known focus of infection unrelated to the lower respiratory tract at inclusion, active possible or probable fungal infection at inclusion, or concomitant participation in clinical research in which the subject was exposed to additional radiation. Patients could participate only once in case of multiple febrile episodes.

### Design

After conformation of neutropenic fever the diagnostic workup was started according to the ESMO guidelines [1] and patients received broad spectrum antibiotics (imipenem) within 2 hours. All patients received selective digestive tract decontamination at the start of chemotherapy, comprising of ciprofloxacin 2dd 500 mg and fluconazole 1dd 150 mg. An additional LDCT scan was made within 24 hours after the start of fever for research purposes only. All LDCT scans remained blinded during the study. The study patients were monitored until they were 24 hours without fever. After this follow-up period and when all culture results were available a predefined consensus diagnosis of the cause of the fever episode was made by an expert panel consisting of 2 haematologists, a microbiologist and a radiologist.

#### Diagnostic workup for febrile neutropenia

The diagnostic workup included a clinical examination, a 2-view CXR and microbiological screening. Standard microbiological screening included evaluation of urine and blood cultures.[1, 4] A respiratory microbiological evaluation was performed in case of a suspected pulmonary infection (clinical symptoms of dyspnea or cough, sputum production, or an oxygen saturation level <93%). For the respiratory evaluation a throat swab was taken and tested for viral pathogens with PCR pack 1 (influenza virus, respiratory syncytial virus (RSV), coronavirus, rhinovirus) and PCR pack 2 (adenovirus, human metapneumovirus (HMPV), parainfluenzavirus type 1–4, bocavirus and *Mycoplasma pneumoniae*). In case of a possible atypical pneumonia the swab was additionally tested for atypical pathogens with PCR pack 3 (*Legionella pneumoniae*, *Chlamydophila pneumoniae*, *Chlamydophila psitacci*, *Coxiella burnetti*). If sputum was available this was tested for bacteria (gram stain, culture) and fungal pathogens (blancophore stain, culture). Other microbiological testing was only performed if judged appropriate by the attending physician. Furthermore, serum samples were taken twice weekly for galactomannan (GM) testing at the end of inclusion.

If fever persisted for 4 days, a routine HRCT scan was acquired to detect possible IFD. In case of abnormalities suspect for (fungal) pulmonary infection a broncho-alveolar lavage (BAL) was performed. BAL fluid was routinely tested for bacteria (gram, stain, culture) including Haemophilus influenza, and fungal pathogens (blancophore stain, GM, microscopy and culture). On indication BAL fluid was also tested for *Nocardia* (culture), Mycobacteria (culture, microscopy, PCR), Pneumocystis jiroveci pneumonia (PCR, microscopy) and viral pathogens: PCR pack 1,2 and 3. In case of IFD anti-fungal treatment was started and a HRCT was repeated after six weeks.

#### Consensus diagnosis

The consensus diagnosis of a fever episode was defined according to predefined categories as either pulmonary infection or non-pulmonary causes of fever such as line infection, mucositis, other infections (i.e. sinusitis), unknown focus of fever or any combination of the above. The expert panel diagnosis was based on information obtained from the clinical charts, microbiology results and imaging results: the 2-view CXR and the HRCT. HRCT was only available in case of persistent (4 days) fever, and LDCT results were not used for the consensus diagnosis.

Clinical criteria for a pulmonary infection included either one of the following symptoms: coughing, sputum production, dyspnea or an oxygen saturation level <93%. Microbiological criteria were a positive culture or PCR for bacterial, viral and fungal pathogens known to cause pulmonary infections, or in case of invasive aspergillosis a positive GM in BAL (≥0.8) or serum (≥0.5). In case of abnormal imaging results without additional clinical or microbiological criteria these were considered false positive. Fungal infections were classified as either possible, probable or proven in accordance with the revised European Organization for Research and Treatment of Cancer/Mycosis Study Group (EORTC/MSG) criteria.[12] IFD was considered to be ruled out in all patients recovering from neutropenic fever within 3 days without ever receiving mould-active antifungal therapy, given the very low likelihood of spontaneous recovery during neutropenia, without appropriate treatment.

Central venous catheter infections were defined by criteria adapted from the Dutch surveillance network of nosocomial infections (PREZIES).[13]

Mucositis was diagnosed based on clinical and radiological (typhlitis) criteria. Clinical criteria included pain when swallowing, nausea, vomiting, abdominal pain/cramping and diarrhea, physical examination and the absence of a positive microbiological test.

Any other focus of infection was mainly determined by the judgment of the attending physician combined with the results of the diagnostic workup. Fever of unknown origin was defined as fever without any focus or etiology identified by clinical, radiological or microbiological examination.

### Image acquisition and analysis

The LDCT scan was made during inspiration at the lowest achievable radiation dose. LDCT images were acquired at a tube potential of 80 kVp and a tube current-time product of 10 mAs or 20 mAs depending on patient’s weight using a 256-slice CT system (iCT, Philips Healthcare, Best, The Netherlands), or at a tube current-time product of 15 mAs or 30 mAs with a 64-slice CT system (Brilliance 64, Philips Healthcare, Best, The Netherlands). At the end of the study, anonymized CXR and LDCT images were first independently evaluated by a board certified radiologist and a radiology resident who were not involved in the consensus diagnosis. The observers used a standard imaging scoring table (Table 1). Second, CXR and LDCT images were re-analysed if one or two observers gave a positive score. Re-evaluation was performed during consensus reading of the study exams, for which additional historical radiological images were available to prevent false-positives as a result of pre-existing abnormalities.

**Table 1:** Imaging scoring table.

Signs	CXR	LDCT
Consolidation	Y/N	Y/N
Subpleural	Y/N	Y/N
Atelectasis	Y/N	Y/N
Ground-glass area(s)		Y/N
Nodule(s) / Mass		Y/N
Size ≥ 10 mm		Y/N
Solid		Y/N
Ground-glass		Y/N
Halo sign*		Y/N
Multiple nodules		Y/N
Tree-in-bud sign		Y/N
Air-crescent sign*		Y/N
Cavity*		Y/N
Pleural effusion	Y/N	Y/N
Unilateral	Y/N	Y/N
Bilateral	Y/N	Y/N
Pericardial effusion	Y/N	Y/N
Interlobular septal thickening	Y/N	Y/N
**Diagnosis**		
Pneumonia	Y/N	Y/N
Lobar	Y/N	Y/N
Broncho	Y/N	Y/N
Interstitial	Y/N	Y/N
Bronchiolitis	Y/N	Y/N
Suspect for fungal infection	Y/N	Y/N

* Signs suspect for fungal infection; Y: yes, finding present, N: no, finding absent.

### Statistical analysis

The primary objective was to investigate whether LDCT is a better diagnostic tool than CXR for detection of pulmonary infections on the first day of neutropenic fever. We hypothesized that LDCT could increase the detection rate of pulmonary infiltrates by 80%; from 15% with CXR (unpublished data from our institute) to 27%, considering an expected incidence of pulmonary focus of infection in febrile neutropenia of 30%.[14] We expected a proportion of discordant pairs of 15% with a two-sided test (alpha 0.05 and power 0.80). A sample size of 68 patients was esteemed sufficient according to a power calculation based on the McNemar test.

Sensitivity, specificity, positive predictive value (PPV) and negative predictive value (NPV) of both tests were calculated using contingency tables, the consensus diagnosis served as reference standard. An uncorrected McNemar test was applied to determine the differences between the two modalities.[15] A *p*-value of ≤0.05 was considered significant. Parametric data are presented as means ± standard deviation (SD) and non-parametric data as medians (range). Cohen’s Kappa (κ) was used to assess the inter observer agreement and defined as excellent with κ > 0.80, good with κ between 0.61 and 0.80, moderate with κ between 0.41 and 0.60, and poor with κ ≤ 0.40. IBM SPSS statistics software version 22.0 (IBM, Somers, NY, USA) was used for statistical analyses.

The secondary objective was to explore the performance of LDCT for detection of pulmonary lesions indicating IFD on day 1 of neutropenic fever.

## Results

**Fig 1:**
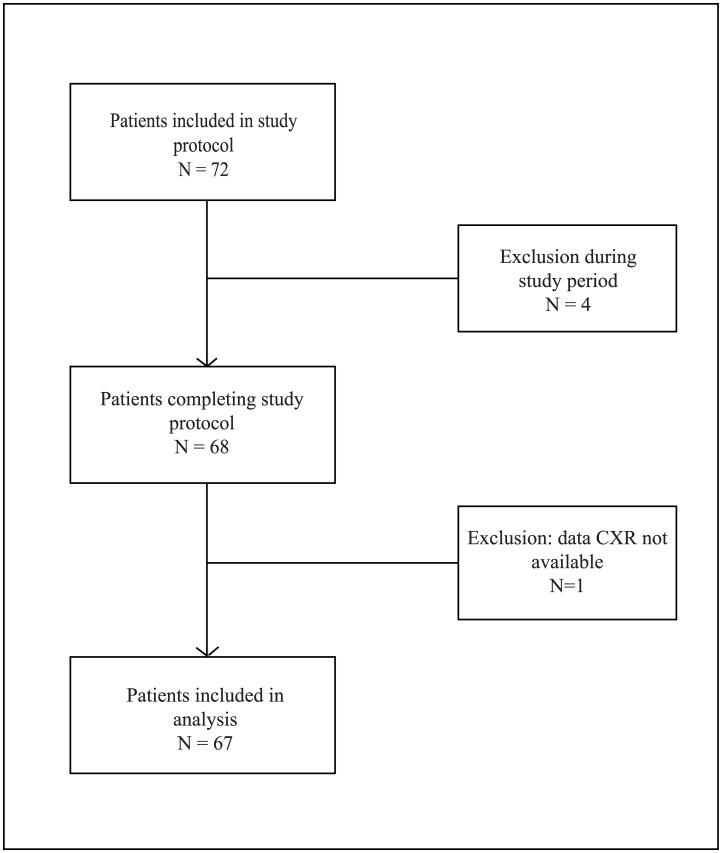
Flowchart.

A total of 72 consecutive patients were recruited between March 2013 and December 2014. Five patients were excluded: one because of a known infection unrelated to the respiratory tract at inclusion, one as a result of active fungal infection, one because the LDCT was acquired more than 24 hours after the CXR, one was not able to undergo LDCT scanning and for one patient CXR was not available (Fig 1). Baseline characteristics of the 67 patients are listed in Table 2.

**Table 2:** Baseline characteristics (n = 67).

Characteristic		No.	%
Gender			
	Male	47	(70.1)
	Female	20	(29.9)
Age (years)			
	Median (range)	58.5	(23–74)
BMI (kg/m^2^)			
	Mean (SD)	24.6	(3.3)
Haematological disease			
	AML/MDS	30	(44.8)
	ALL	3	(4.5)
	NHL	8	(11.9)
	Multiple Myeloma	18	(26.9)
	Myelofibrosis	4	(6.0)
	M. Hodgkin	2	(3.0)
	Systemic Sclerosis	2	(3.0)
Therapeutic modality			
	Induction chemotherapy	26	(38.8)
	Allogeneic SCT	15	(22.4)
	HDM + autologous SCT	16	(23.9)
	BEAM + autologous SCT	3	(4.5)
	Other	7	(10.4)
Neutropenic episode (days)			
	Median (range)	13.5	(3–68)
Days of neutropenia until fever			
	Median (range)	5	(0–51)
Fever (days)			
	Median (range)	4	(1–46)

SD = standard deviation; AML = acute myeloid leukaemia; MDS = myelodysplastic syndrome; ALL = acute lymphocytic leukaemia; NHL = non Hodgkin lymphoma; SCT = stem cell transplantation; HDM = high-dose melphalan; BEAM = carmustine, etoposide, cytarabine and melphalan.

According to the consensus diagnosis 11 patients (16.4%) had a pulmonary infection, of which 5 had IFD (2 possible, 3 probable), 1 had rhinovirus and 5 pneumonia of unknown aetiology. Five of the patients with pneumonia underwent bronchoscopy due to persistent fever and abnormalities on HRCT. In 3 of those patients a probable pathogen was identified after evaluation of BAL-fluid, which was probable IFD in all 3.

Furthermore in 35 (55.2%) patients, overall nonspecific mucositis was the most likely cause of fever. In four of these patients abdominal imaging (CT) was performed showing no signs of typhlitis. In 15 patients (22.4%) a focus of infection was not found. The other consensus diagnosis results are listed in Table 3.

**Table 3:** Consensus diagnosis neutropenic fever episodes (n = 67).

Consensus Diagnosis			No.	(%)
Pulmonary Infection			11	(16.4)
	Bacterial Pneumonia		0	
	Viral Pneumonia		1	(1.5)
	Fungal Pneumonia		5	(7.5)
		Possible	2	(3.0)
		Probable	3	(4.5)
		Proven	0	
	Unknown pathogen		5	(7.4)
Central venous catheter infection			3	(4.5)
Mucositis			35	(55.2)
Miscellaneous causes			16	(23.9)
Unknown			15	(22.4)
**Total**			80^a^	

^a^In 13 patients multiple causes of fever were described in a single fever episode.

### Imaging results

The mean interval between CXR and LDCT was 3.1 ± 6.3 hours. The mean radiation dose for LDCT exams was 0.24 ± 0.15 mSv, which is comparable to 2–3 chest radiographs (average dose posteroanterior and lateral chest radiograph: 0.1 mSv).[9] None of the patients experienced adverse events as a result of LDCT scanning or chest radiography. Eight CXRs (11.9%) were indicative of pneumonia, 4 CXRs were false positive and 7 false negative as compared to the consensus diagnosis (Table 4). This resulted in a sensitivity of 36%, a specificity of 93%, a PPV of 50% and a NPV of 88% for CXR. None of the CXRs were suspect for IFD.

**Table 4:** Contingency tables for CXR and LDCT results as compared to the consensus diagnosis.

**Pulmonary Infection**		**Yes (n = 11)**	**No (n = 56)**	
CXR	Positive (n = 8)	4	4	**PPV 50% (17–83%)**
	Negative (n = 59)	7	52	**NPV 88% (76–95%)**
		**Sensitivity 36% (12–68%)**	**Specificity 93% (82–98%)**	
**Pulmonary Infection**		**Yes (n = 11)**	**No (n = 56)**	
LDCT	Positive (n = 13)	8	5	**PPV 62% (32–85%)**
	Negative (n = 54)	3	51	**NPV 94% (84–99%)**
		**Sensitivity 73% (39–93%)**	**Specificity 91% (80–97%)**	

CXR = chest X-ray; LDCT = low-dose computed tomography; PPV = positive predictive value; NPV = negative predictive value; (…) = confidence interval. Uncorrected McNemar test: *p* = 0.197

Thirteen LDCT scans (19.4%) were suggestive of pneumonia. Compared to the consensus diagnosis, 5 LDCT scans were false positive and 3 false negative. Sensitivity, specificity, PPV and NPV were 73%, 91%, 62% and 94% respectively. (Table 4)

Consensus diagnosis of the 5 false positive LDCT scans were phlebitis and in 4 cases fever of unknown origin. No HRCT scans were made because in all 5 patients the fever subsided within 48 hours after receiving broad spectrum antibiotics, therefore CXR was the only available imaging tool for the consensus meeting. Only one of the patients with a false positive LDCT had respiratory symptoms (cough), however a microbiological respiratory evaluation was negative.

An uncontrolled McNemar’s test showed no statistically significant difference in the proportion of scans positive for pulmonary infection between CXR and LDCT (*p* = 0.197), which was our primary endpoint. Inter-observer agreement was moderate for both CXR and LDCT (κ = 0.52 for CXR and κ = 0.52 for LDCT, respectively).

**Fig 2:**
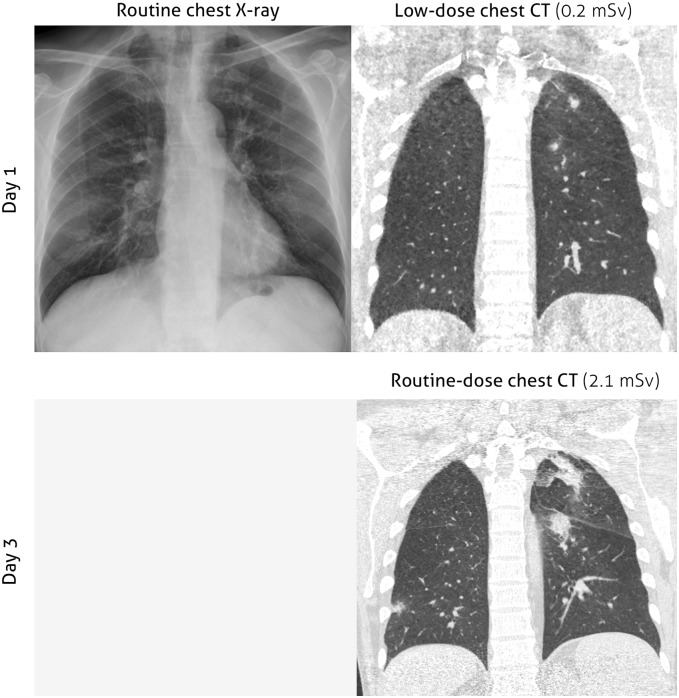
Patient with a positive LDCT scan for fungal infection on day 1 of neutropenic fever. Upper row left: CXR acquired on day 1 of febrile neutropenia without signs of pulmonary infection. Upper row right: LDCT images showing solid consolidations with halo signs suspect for IFD at day 1 of febrile neutropenia. Lower row: HRCT acquired on the 3^rd^ day of neutropenic fever shows progression of the consolidations and new consolidations.

Based on the consensus diagnosis 5 patients in this cohort were classified as either probable (n = 3) or possible (n = 2) IFD. The diagnosis of probable IFD was based on a positive BAL GM test in all cases and 2 out of 3 also had a positive serum GM test. Only 1 patient had a positive BAL culture (*A*. *Versicolor)*. Four of the patients with IFD (3 probable, 1 possible) had a LDCT scan suspect for IFD on the first day of fever. In all these patients the abnormalities were also seen on the HRCT performed as part of the diagnostic workup for persistent fever (mean 3.5 days later than LDCT). (Fig 2) None of the patients diagnosed with pulmonary IFD had abnormalities on their CXR on the first day of fever, and only one had respiratory signs or symptoms.

The diagnosis of possible IFD in the patient with a negative LDCT scan was based on abnormalities on HRCT made on day 4 of fever. Serum GM values were negative and bronchoscopy was not performed because of severe thrombocytopenia. Finally one LDCT scan with abnormalities suspect for IFD was considered false positive: the fever episode was classified as “fever of unknown origin”.

## Discussion

We conducted a prospective study to evaluate whether pulmonary focus detection would improve using a LDCT scan instead of CXR on the first day of febrile neutropenia. We established an improved detection rate from 11.9% of radiographs to 19.4% of the LDCT scans but our primary endpoint was not met. As a result of the lower than expected incidence of pulmonary infections in this study, the study was underpowered.[14] Nevertheless, we demonstrated a clinically significant increased sensitivity of LDCT (73% versus CXR 36%) in detecting a pulmonary focus on day 1 of neutropenic fever.

The limited value of CXR as a standard diagnostic procedure is in line with previous results.[3] In a retrospective study 1083 adult SCT patients were evaluated, but in none of the 242 CXRs performed in asymptomatic patients with febrile neutropenia pulmonary abnormalities indicative of infection were detected. In contrast, in 76 patients with respiratory symptoms 24 CXRs showed evidence of pneumonia.[3] In our study 4 patients had a true positive CXR; 1 of these patients had respiratory symptoms on the first day of fever.

Two previous studies have compared the use of CXR with LDCT in febrile neutropenic patients. In the present study a much lower mean radiation dose of 0.24 mSv was used compared to 0.6 mSv and 1.5 mSv in the other studies.[10, 11] Still, reconstructed images were of diagnostic quality in all patients. The LDCT scans in the previous studies were acquired using older generation CT systems (4 slice CT scanner by Patsios *et al*.[10] and 16 slice CT scanner by Kim *et al*.[11].) We used two newer generation CT systems (64 slices and 256 slices) with newer X-ray tubes, detectors and software, resulting in the potential to further reduce the radiation dose without compromising on image quality.

Both previous studies demonstrated that LDCT increased the detection rate but they differ in several aspects from our study. Patsios *et al*.[10] compared CXR with LDCT in neutropenic AML patients with already clinically suspected pneumonia, and found abnormal imaging results in 31 of 40 CXRs, whereas 38 out of 40 patients had abnormalities on LDCT. Instead of only focusing on pulmonary abnormalities suggestive of pulmonary infection, this study also included radiological signs of cardiac failure or fluid overload, which might explain the high number of patients with abnormalities in both CXR and LDCT.[10]

In a prospective study Kim *et al*.[11] evaluated the use of LDCT in a selection of patients with persistent neutropenic fever (>48 hours) regardless of the presence of respiratory symptoms. Of the 207 included patients 150 were diagnosed with pneumonia (72%). Differences between sensitivity of CXR and LDCT for correctly diagnosing infectious pneumonia were a little less pronounced than in our study: CXR 39% and LDCT 63%.

To our knowledge this is the first study to evaluate LDCT in all patients with febrile neutropenia on day 1 of fever. We demonstrated an increase in sensitivity and an improved NPV when performing LDCT in the detection of a pulmonary infection in febrile neutropenia. This is important because detection of a focus of infection in neutropenic fever is difficult and patients often undergo several diagnostic tests and uncertain treatments which can have negative side effects. Therefore it can be expected that every improvement in the diagnostic workup will eventually lead to improved patient care.

A major advantage of our study is the completely independent assessment of both index and reference test (LDCT and CXR by independent radiologists) and the short time interval between CXR and LDCT (3.1 ± 6.3 hours). However, the inter-observer agreement was moderate and should be improved when moving forward with the LDCT technique. This points out that evaluation of LDCT images in this neutropenic population at high-risk of developing pulmonary infections requires expert-thoracic radiologists, which may not yet be available in every hospital.

A limitation of the study is the use of the reference standard (consensus diagnosis) which may have contributed to the lower test performance of LDCT. In 38 of the 67 patients a CXR was the only imaging modality available for the consensus meeting, since HRCT was only performed in case of persistent fever. Considering the low sensitivity of CXR in this population, pulmonary abnormalities could have been missed, This may have led to an underestimation of the amount of patients with pneumonia, and LDCTs might have incorrectly be judged as false positives.

Furthermore clinical symptoms of cough, sputum production and dyspnea are not always evident in a (bedridden) neutropenic patient.[16] An extensive microbiological evaluation for respiratory causes of fever, which was only performed upon clinical indication, might therefore not have been performed in all patients that had abnormalities suspect for pulmonary infection on LDCT. Several studies report on the limited diagnostic yield of a respiratory microbiological evaluation in neutropenic patients. In up to 70% of the lower airway infections a pathogen can not be identified.[17, 18] Sputum cultures (which are often not available) only reveal a possible pathogen in less than 50% of cases.[18] BAL seems to be the diagnostic procedure with the highest yield. Despite the low detection rates of a probable pathogen in approximately 50% of cases, it is the most sensitive procedure for detecting IFD.[19] However, since routinely performance of BAL in the initial assessment of fever is not performed due to its invasive nature, a consensus diagnosis as was used in our study is the best available option and complies with established guidelines.[1, 4]

We were able to identify a probable pathogen in 36% of the patients with pneumonia (2 cases of possible IFD excluded), which is consistent with reports in literature.[17, 18] However we did not establish any case of bacterial pneumonia. This may be explained by the low amount of patients with sputum available for culture (36%, all culture-negative), or a possible treatment response to broad spectrum antibiotics, resulting in defervescence within 3 days, in which case HRCT and BAL were not performed.

We included a heterogenic population with patients with prolonged neutropenia as well as patients with shorter neutropenic episodes. This could have had an effect on the incidence of pulmonary infections, because the risk of developing pneumonia (especially IFD) increases in case of prolonged neutropenia.

The use of LDCT scans for detection of pulmonary lesions indicating IFD at day 1 of neutropenic fever seems promising. Out of 5 fever episodes classified as either possible or probable IFD, 4 patients already had abnormalities suspect for IFD on their LDCT whereas these where not seen on CXR. Importantly, only one of these patients had respiratory signs or symptoms and therefore we think that omitting chest imaging in patients without respiratory symptoms can lead to a delayed diagnosis of IFD.

Incorporation of LDCT in the diagnostic workup of all patients with neutropenic fever will increase costs when compared to CXR. However, initiation of early and targeted treatment of IFD may reduce overall costs, for example by reducing length of hospital stay, intensive care unit admittance rates and the amount of diagnostic procedures required. Therefore the performance of LDCT in all patients with febrile neutropenia might still be cost-effective. This issue should be evaluated in future research.

## Conclusion and implication

Performance of CXR in the initial assessment of febrile neutropenia is of limited value for detection of pulmonary abnormalities. The introduction of LDCT improved the detection of pulmonary infiltrates and there was a clear signal that LDCT scanning is capable of detecting invasive fungal infections at a very early stage. Therefore, the use of LDCT in the initial assessment of febrile neutropenia is promising, and should be further evaluated in a larger study powered on IFD detection. Furthermore it would be interesting to see whether LDCT could replace HRCT as imaging tool in patients with suspected IFD in order to decrease radiation exposure.
